# Potentially modifiable factors to reduce severity of COVID-19 in type 2 diabetes

**DOI:** 10.1038/s41387-020-00133-0

**Published:** 2020-08-12

**Authors:** Nikhil V. Dhurandhar, Md Akheruzzaman, Vijay Hegde

**Affiliations:** Department of Nutritional Sciences, Texas Tech University, Lubbock, TX 79409 USA

**Keywords:** Biochemistry, Cell biology

## COVID-19 pandemic, diabetes, and obesity

Since December 2019, the severe acute respiratory syndrome coronavirus 2 (SARS-CoV-2 or CoV-2) disease (COVID-19) pandemic has claimed over 517,610 lives worldwide, among which 128,184 are from the US^1^. The appearance, severity, prognosis, and case fatality of COVID-19 is disproportionately higher in people with pre-existing obesity, type 2 diabetes (T2D) and other cardio-metabolic diseases, and in people of racial and ethnic minorities^2^. Furthermore, COVID-19-related damage of the heart and kidneys is greater in the presence of T2D^3–5^, which itself is a major risk factor for cardiovascular disease^6^ and end-stage renal disease (CKD)^7,8^. This is of serious concern for the United States, where two out of three adults have higher body weight, and ~42% have obesity, and the prevalence of obesity is 85% and 30–60% in T2D and hypertension, respectively^9^.

Without effective vaccines or therapies for the prevention or treatment of CoV-2 infection at this time, there is an urgent need for strategies to at least minimize COVID-19-related comorbidities or fatalities. In particular, considering the disproportionately higher COVID-19-related mortality in people with obesity, T2D, and other cardio-metabolic diseases, we wish to share a viewpoint about the possible steps to take, which may save lives.

It is unclear why COVID-19 causes disproportionately more severe symptoms and complications in people with obesity and those with hypertension or T2D. The synergistic stress of acute respiratory distress syndrome and pre-existing obesity and its comorbidities may provide a likely explanation. Here, we wish to outline a possibility related to the negative interaction of CoV-2 and certain anti-diabetic medications, that deserves serious consideration.

The selective high-risk of COVID-19 with obesity, may stem from its very fundamental mechanism of infection. CoV-2 exploits the angiotensin-converting enzyme (ACE)-2 receptor for cellular entry^10,11^. Overexpression of ACE2 can facilitate CoV-2 entry and subsequent severity of infection^12^. CoV-2 virus has a particular affinity for lung cells (airway epithelial cells)^12^. Besides the lung cells, ACE2 is expressed in many cell types, including adipocytes, renal cells, and cardiomyocytes. Although people with obesity do not overexpress ACE2^13^, more cells with ACE2 will be present in individuals with obesity (greater number of adipocytes), which may support greater virus replication and infection. This may be a reason for worse outcome of CoV-2 infection for people with obesity. Intuitively, fat loss appears as an approach to counter worse outcome of CoV-2 infection in obesity. However, a quick induction of fat loss is less likely in COVID-19 patients. Instead, it may be more pragmatic to focus on comorbidities of obesity such as T2D during COVID-19.

At least three key factors that distinguish individuals with obesity and T2D from individuals without such conditions are, greater circulating levels of glucose, inflammatory cytokines, and the use of anti-diabetic drugs. These factors are linked with ACE2 expression or activity, which may in turn, influence the severity of CoV-2 infection. High blood glucose alone can increase the expression and enzymatic activity of ACE2 in cells^14–16^. Inflammatory cytokines like tumor necrosis factor-α, interferon-γ, or interleukin-6, are increased in T2D. It is unclear if these cytokines facilitate CoV-2 entry or oppose it, as they have been reported to increase^17^ or decrease ACE2 expression^18^. In addition, the increased risk of COVID-19 for people with T2D may in part, be iatrogenic. Some anti-diabetic drugs such as those from groups, including PPARγ agonists, SGLT2 inhibitors or GLP1 receptor agonists upregulate ACE2 expression in rodent model or cells, whereas some do not (metformin, insulin)^19^ or have not yet been tested for their effect on ACE2. The drugs that upregulate ACE2 expression may inadvertently facilitate CoV-2 infection in humans, leading to an increase in disease severity and consequential mortality. Some reports suggest the use of PPAR-γ agonist agents, such as pioglitazone in COVID-19 patients to reduce pulmonary fibrosis and inflammation^20,21^. Although, the anti-fibrotic and anti-inflammatory properties of PPAR-γ agonists are attractive, their role in ACE2-mediated entry of CoV-2 needs further examination. Based on the current information, we postulate that in T2D, the role of these three factors should be studied for their individual and/or collective effect on ACE-2 expression, binding of CoV-2 to the receptor, and COVID-19 severity.

## Guidance needed

Owing to the urgency for information, there is a scramble to issue clinical guidelines based on observational information, for the management of hyperglycemia, inflammation, and use of anti-diabetic medication during CoV-2 infection for T2D patients with other comorbidities^22^. However, experimental, cell-based or animal evidence to guide these clinical decisions is needed.

This is particularly important as the interaction of a drug may be different in the presence of CoV-2 infection. For example, increasing ACE2 expression by a therapeutic agent may be desirable in T2D management^23^, but may be detrimental in the presence of COVID-19. Hydroxychloroquine is an example of why experimental evidence is necessary, even for intuitive sounding solutions. It is a normally useful drug for malaria, that was highly touted for COVID-19 treatment, but failed to treat COVID-19^24,25^, and in fact increased mortality in the presence of CoV-2 infection^26^.

Generating underlying mechanistic evidence can increase confidence in the accuracy of these guidelines or may provide the opportunity for a course correction. For example, the worse outcome of COVID-19 in diabetes may in part be of iatrogenic origin. Perhaps, the drugs that upregulate ACE2 expression, inadvertently facilitate CoV-2 infection, leading to greater severity and mortality. Identification of anti-diabetic drugs that do not promote ACE-2 expression or increase COVID-19 severity may provide better clinical choices for treating T2D patients with COVID-19. Similarly, COVID-19 invokes a response of inflammatory cytokines, known as cytokine storms^27^. Clinical efforts are directed towards reducing this systemic inflammatory response. However, it would be important to determine the effect of cytokine storm or its treatment on ACE-2 expression and CoV-2 binding.

## Conclusions

Severity and mortality of COVID-19 caused by the CoV-2 virus, is greater in people with obesity, hypertension, and diabetes. While there may be many known or unknown factors contributing to this phenomenon, here we focus on one specific possibility. Worse outcome of COVID-19 in diabetes may in part be of iatrogenic origin. Angiotensin-converting enzyme-2 (ACE2) acts as a receptor for cellular entry of CoV-2. Some, but not all anti-diabetic drugs upregulate ACE2 expression, which further can be modulated in the presence of high level of glucose and inflammatory cytokines. Perhaps, the drugs that upregulate ACE2 expression, inadvertently facilitate CoV-2 infection, leading to greater severity and mortality. We urge research to identify the anti-diabetic drugs that do or do not facilitate CoV-2 entry in cells, and to determine their anti-diabetic efficacy in COVID-19. This could help replace anti-diabetic medications that promote CoV-2 entry in cells with alternatives that do not, and yet are effective. While the obesity status of COVID-19-infected individuals cannot be changed rapidly, medications for their diabetes management can promptly be switched to an optimal option, which may save many lives.
